# Supplementary material for: Interdigital and Plantar Foot Infections: A Retrospective Analysis of Molecularly Diagnosed Specimens in the United States and a Literature Review
Source: Microorganisms. 2025 Jan 16;13(1):184. doi: 10.3390/microorganisms13010184 (PMC11767718; doi:10.3390/microorganisms13010184)
Supplement: Supplementary file 1 [file microorganisms-13-00184-s001.zip › microorganisms-3392471-supplementary.pdf]

Interdigital and plantar foot infections: A retrospective analysis of molecularly diagnosed specimens in the United States and literature review

Aditya K. Gupta, Tong Wang, Sara A. Lincoln, and Wayne L. Bakotic

Microorganisms

Supplementary Materials

Table of Contents

Table S1. 2
References..4

Table S1. Literature review of studies identifying fungal and/or bacterial etiological agents in patients with foot infections (2010-2024), stratified per the sampling site.

Study ID	Region	Population[a] (N)	Identification Method	Fungal Agents[b]	Bacterial Agents[b]
**Interdigital foot infection**					
Diongue 2020 [1]	Senegal	General (169)	Culture, MALDI-TOF	*C. albicans*, *Fusarium* spp., *T. interdigitale*, *T. rubrum*, *Epidermophyton floccosum*[c], *C. tropicalis*[c], *C. fermentati*[c]	-
Abd Elmegeed 2015 [2]	Egypt	General (135)	Culture	*T. mentagrophytes*, *T. rubrum*, *T. interdigitale*	-
Sariguzel 2014 [3]	Turkey	General (121)	Culture	*T. rubrum*, *T. mentagrophytes*, *C. albicans*, *Trichosporon* spp., *C. glabrata*[c], *Aspergillus* spp[c]., *Fusarium* spp.[c], *Acremonium* spp.[c], *Rhodotorula* spp.[c]	*C. minutissimum*
Diongue 2016 [4]	Senegal	General (104)	Culture	*C. albicans*, non-*albicans Candida*, *Fusarium solani*, *T. interdigitale*, *F. oxysporum*	-
Zarrin and Babadi 2014 [5]	Iran	Pediatric (865) (*asymptomatic*)	Culture	*Penicillium* spp., *Rhizopus* spp., *Alternaria* spp., *Cladosporium* spp., *Aspergillus flavus*	-
Saud 2020 [6]	Nepal	Diabetic (67)	Culture	*C. albicans*, *C. glabrata*[c], *Trichophyton* spp.[c], *C. krusei*, *Aspergillus flavus*	-
		Non-Diabetic (67)	Culture	*Aspergillus flavus*, *A. niger*[c], *C. albicans*[c], *C. glabrata*[c],	-
Thimmappaiah Jagadeesh 2017 [7]	India	Diabetic (40)	Culture	*Candida* spp.	*S. epidermidis*, *Micrococcus* spp., *S. aureus*, *Corynebacterium* spp., *Bacillus* spp.
		Non-Diabetic (41)	Culture	*Candida* spp.	*Micrococcus* spp., *S. epidermidis*, *Bacillus* spp., *S. aureus*[c], *Corynebacterium*[c]
**Plantar foot infection**					
Sakka 2015 [8]	Israel	General (221) (*asymptomatic*)	Culture	*T. rubrum*, *T. mentagrophytes*[c], *Epidermophyton floccosum*[c]	-
**Sampling site unspecified**					
Yu 2020 [9]	China	General (1704)	Culture, sequencing	*T. rubrum*, *T. interdigitale*, non-*albicans Candida*, *C. albicans*, *Epidermophyton floccosum*	*S. epidermidis*, *S. aureus*, *S. haemolyticus*, *S. warneri*, *S. aureus* subsp. *aureus*.

Mäntyniemi 2024 [10]	Finland	General (295)	Culture, PCR	*T. rubrum, T. interdigitale, T. mentagrophytes, Epidermophyton floccosum*[c], *C. albicans*[c]	-
Chadeganipour 2016 [11]	Iran	General (139)	Culture	*T. mentagrophytes* var. *interdigitale, T. rubrum, Epidermophyton floccosum*	-
Toukabri 2017 [12]	Tunisia	General (78)	Culture	*T. rubrum, T. violaceum, T. verrucosum*	-
Pandit 2017 [13]	India	General (68)	Culture	*T. rubrum, T. mentagrophytes, T. schoenleinii, T. verrucosum*	-
Zareshahrabadi 2020 [14]	Iran	General (62)	Culture, sequencing	*T. mentagrophytes, T. tonsurans, Microsporum canis, T. interdigitale, T. rubrum*	-
Thakur 2015 [15]	Botswana	General (56)	Culture	*T. interdigitale, T. tonsurans*[c], *T. violaceum*[c]	-
Liu 2019 [16]	China	General (26)	Metagenomic sequencing	*T. rubrum, C. halotolerans, C. parapsilosis, Wallemia sebi*	*C. tuberculostearicum, S. pettenkoferi, C. minutissimum, Paracoccus sphaerophysae*
		General (10) (*asymptomatic*)	Metagenomic sequencing	*Phoma saxea, Aspergillus cibarius, C. halotolerans, Rhodotorula mucilaginosa*	*C. tuberculostearicum, S. pettenkoferi, Pseudoclavibacter alba, Brevibacterium paucivorans*
Bitew 2018 [17]	Ethiopia	General (14)	Culture	*T. mentagrophytes, T. rubrum, T. tonsurans*[c], *T. soudanense*[c], *Alternaria* spp.[c], *Fusarium* spp.[c]	-
Hazarika 2019 [18]	India	General (9)	Culture	*T. mentagrophytes*	-
Makwana 2020 [19]	India	General (7)	Culture	*T. rubrum*	-
Taha 2017 [20]	Egypt	General (5)	Culture	*T. mentagrophytes*	-
Balci 2014 [21]	Turkey	Pediatric (41)	Culture	*T. rubrum, Rhodotorula* spp., *C. glabrata, Trichosporon* spp., *T. mentagrophytes*[c], *C. albicans*[c]	-
Leibovici 2014 [22]	Israel	Psoriasis (232)	Culture	*T. rubrum, T. mentagrophytes, C. albicans, Scopulariopsis brevicaulis*	-
		Atopic Dermatitis (190)	Culture	*T. rubrum, T. mentagrophytes, C. albicans*	-
		General (202)	Culture	*T. rubrum, T. mentagrophytes, C. albicans*	-

[a] General population refers to patients of all ages with and without comorbidities

[b] The five most commonly detected fungal or bacterial agents are listed in decreasing order

[c] Detected in equal proportions

References

1. Diongue, K.; Samb, D.; Seck, M.C.; Diallo, M.A.; Ndiaye, M.; Faye, M.D.; Badiane, A.S.; Ranque, S.; Ndiaye, D. Use of MALDI-TOF MS for Fungal Species Distribution of Interdigital Intertrigo in Seafarers, Dakar, Senegal. *J. Mycol. Med.* **2020**, *30*, 100974, doi:10.1016/j.mycmed.2020.100974.

2. Abd Elmegeed, A.S.M.; Ouf, S.A.; Moussa, T.A.A.; Eltahlawi, S.M.R. Dermatophytes and Other Associated Fungi in Patients Attending to Some Hospitals in Egypt. *Brazilian J. Microbiol.* **2015**, *46*, 799–805, doi:10.1590/S1517-838246320140615.

3. Sariguzel, F.M.; Nedret Koc, A.; Yagmur, G.; Berk, E. Interdigital Foot Infections: *Corynebacterium Minutissimum* and Agents of Superficial Mycoses. *Brazilian J. Microbiol.* **2014**, *45*, 781–784, doi:10.1590/S1517-83822014000300003.

4. Diongue, K.; Ndiaye, M.; Diallo, M.A.; Seck, M.C.; Badiane, A.S.; Diop, A.; Ndiaye, Y.D.; Déme, A.; Ndiaye, T.; Ndir, O.; et al. Fungal Interdigital Tinea Pedis in Dakar (Senegal). *J. Mycol. Med.* **2016**, *26*, 312–316, doi:10.1016/j.mycmed.2016.04.002.

5. Zarrin, M.; Babadi, N. Mycoflora of the Interdigital Spaces among Girl Students in Ahvaz, Iran. *Jundishapur J. Microbiol.* **2014**, *7*, 10–12, doi:10.5812/jjm.12188.

6. Saud, B.; Bajgain, P.; Paudel, G.; Shrestha, V.; Bajracharya, D.; Adhikari, S.; Dhungana, G.; Awasthi, M.S. Fungal Infection among Diabetic and Nondiabetic Individuals in Nepal. *Interdiscip. Perspect. Infect. Dis.* **2020**, *2020*, doi:10.1155/2020/7949868.

7. Thimmappaiah Jagadeesh, A.; Prakash, P.Y.; Karthik Rao, N.; Ramya, V. Culture Characterization of the Skin Microbiome in Type 2 Diabetes Mellitus: A Focus on the Role of Innate Immunity. *Diabetes Res. Clin. Pract.* **2017**, *134*, 1–7, doi:10.1016/j.diabres.2017.09.007.

8. Sakka, N.; Shemer, A.; Barzilai, A.; Farhi, R.; Daniel, R. Occult Tinea Pedis in an Israeli Population and Predisposing Factors for the Acquisition of the Disease. *Int. J. Dermatol.* **2015**, *54*, 146–149, doi:10.1111/ijd.12506.

9. Yu, J.; Liu, W. Da; Tong, Z.S.; Yu, N.; Cao, C.W.; Zhou, X.; Li, Y.Z.; Zhang, Y.; Li, F.Q.; Zhang, J.M.; et al. Aetiology of Superficial Fungal Infections of the Foot in Urban Outpatients in Mainland China: A Multicentre, Prospective Case Study. *Mycoses* **2020**, *63*, 1235–1243, doi:10.1111/myc.13168.

10. Mäntyniemi, T.; Säntti, S.; Kiviniemi, E.; Jokelainen, J.; Huilaja, L.; Sinikumpu, S.P. Superficial Fungal Infections in Adults in Northern Finland between 2010 and 2021: A Register-Based Study. *Heal. Sci. Reports* **2024**, *7*, 1–9, doi:10.1002/hsr2.70138.

11. Chadeganipour, M.; Mohammadi, R.; Shadzi, S. A 10-Year Study of Dermatophytoses in Isfahan, Iran. *J. Clin. Lab. Anal.* **2016**, *30*, 103–107, doi:10.1002/jcla.21852.

12. Toukabri, N.; Dhieb, C.; El Euch, D.; Rouissi, M.; Mokni, M.; Sadfi-Zouaoui, N. Prevalence, Etiology, and Risk Factors of Tinea Pedis and Tinea Unguium in Tunisia. *Can. J. Infect. Dis. Med. Microbiol.* **2017**, *2017*, doi:10.1155/2017/6835725.

13. Pandit, V.S.; Mehta, H. A Hospital-Based Cross-Sectional Clinicomycological Study of Dermatophytoses in a Tertiary Care Centre. *J. Pakistan Assoc. Dermatologists* **2017**, *27*, 375–380.

14. Zareshahrabadi, Z.; Totonchi, A.; Rezaei-Matehkolaei, A.; Ilkit, M.; Ghahartars, M.; Arastehfar, A.; Motamedi, M.; Nouraei, H.; Sharifi Lari, M.; Mohammadi, T.; et al. Molecular Identification and Antifungal Susceptibility among Clinical Isolates of Dermatophytes in Shiraz, Iran (2017-2019). *Mycoses* **2021**, *64*, 385–393, doi:10.1111/myc.13226.

15. Thakur, R. Spectrum of Dermatophyte Infections in Botswana. *Clin. Cosmet. Investig. Dermatol.* **2015**, *8*, 127–133, doi:10.2147/CCID.S78237.

16. Liu, X.; Tan, J.; Yang, H.; Gao, Z.; Cai, Q.; Meng, L.; Yang, L. Characterization of Skin Microbiome in

Tinea Pedis. *Indian J. Microbiol.* **2019**, *59*, 422–427, doi:10.1007/s12088-019-00816-y.

17. Bitew, A. Dermatophytosis: Prevalence of Dermatophytes and Non-Dermatophyte Fungi from Patients Attending Arsho Advanced Medical Laboratory, Addis Ababa, Ethiopia. *Dermatol. Res. Pract.* **2018**, *2018*, doi:10.1155/2018/8164757.

18. Hazarika, D.; Jahan, N.; Sharma, A. Changing Trend of Superficial Mycoses with Increasing Nondermatophyte Mold Infection: A Clinicomycological Study at a Tertiary Referral Center in Assam. *Indian J. Dermatol.* **2019**, *64*, 261–265, doi:10.4103/ijd.IJD_579_18.

19. Makwana, G.E.; Mathur, M. Clinico-Mycological Evaluation of Dermatophytes in a Tertiary Care Hospital of Saurashtra, Gujarat, India. *J. Clin. Diagnostic Res.* **2020**, *14*, 26–29, doi:10.7860/jcdr/2020/43639.13880.

20. Taha, M.; Elfangary, M.; Essa, S.; Younes, A. Species Identification of Dermatophytes Isolated from Human Superficial Fungal Infections by Conventional and Molecular Methods. *J. Egypt. Women's Dermatologic Soc.* **2017**, *14*, 76–84, doi:10.1097/01.EWX.0000499598.84966.cb.

21. Balci, E.; Gulgun, M.; Babacan, O.; Karaoglu, A.; Kesik, V.; Yesilkaya, S.; Turker, T.; Tok, D.; Koc, A.N. Prevalence and Risk Factors of Tinea Capitis and Tinea Pedis in School Children in Turkey. *J. Pak. Med. Assoc.* **2014**, *64*, 514–518.

22. Leibovici, V.; Ramot, Y.; Siam, R.; Siam, I.; Hadayer, N.; Strauss-Liviatan, N.; Hochberg, M. Prevalence of Tinea Pedis in Psoriasis, Compared to Atopic Dermatitis and Normal Controls - A Prospective Study. *Mycoses* **2014**, *57*, 754–758, doi:10.1111/myc.12227.
